# 3D printing method for next-day acetabular fracture surgery using a surface filtering pipeline: feasibility and 1-year clinical results

**DOI:** 10.1007/s11548-019-02110-0

**Published:** 2020-01-02

**Authors:** Simon Weidert, Sebastian Andress, Christoph Linhart, Eduardo M. Suero, Axel Greiner, Wolfgang Böcker, Christian Kammerlander, Christopher A. Becker

**Affiliations:** Department of General, Trauma and Reconstructive Surgery, University Hospital, LMU Munich, Campus Großhadern, Marchioninistr. 15, 81377 Munich, Germany

**Keywords:** Acetabular fracture, 3D printing, 3D model creation, Computer-aided surgery, Pelvic surgery

## Abstract

**Introduction:**

In orthopedic surgery, 3D printing is a technology with promising medical applications. Publications show promising results in acetabular fracture surgery over the last years using 3D printing. However, only little information about the workflow and circumstances of how to properly derive the 3D printed fracture model out of a CT scan is published.

**Materials and methods:**

We conducted a retrospective analysis of patients with acetabular fractures in a level 1 trauma center. DICOM data were preoperatively used in a series of patients with acetabular fractures. The 3D mesh models were created using 3D Slicer (https://www.slicer.org) with a newly introduced surface filtering method. The models were printed using PLA material with FDM printer. After reduction in the printed model, the acetabular reconstruction plate was bent preoperatively and sterilized. A clinical follow-up after 12 months in average was conducted with the patients.

**Results:**

In total, 12 patients included. Mean printing time was 8:40 h. The calculated mean printing time without applying the surface filter was 25:26 h. This concludes an average printing time reduction of 65%. Mean operation time was 3:16 h, and mean blood loss was 853 ml. Model creation time was about 11 min, and mean printing time of the 3D model was 8:40 h, preoperative model reduction time was 5 min on average, and preoperative bending of the plate took about 10 min. After 12 months, patients underwent a structured follow-up. Harris Hip Score was 75.7 points, the Modified Harris Hip Score 71.6 points and the Merle d’Aubigne Score 11.1 points on average.

**Conclusions:**

We presented the first clinical practical technique to use 3D printing in acetabular fracture surgery. By introducing a new surface filtering pipeline, we reduced printing time and cost compared to the current literature and the state of the art. Low costs and easy handling of the 3D printing workflow make it usable in nearly every hospital setting for acetabular fracture surgery.

## Introduction

Additive manufacturing or 3D printing (3DP) is a technology that has shown a significant potential in the medical field with publications increasing by 13-fold since 2014 [1]. Its capability to create almost any three-dimensional form has been used across various specialties and applications [2]. In the field of orthopedics, 3D printed cutting and drilling guides [3], as well as individualized implants, prosthetics and orthotics, have become commercially available [4].

Even though raw printing times of less than a day can be achieved, acquiring data and transforming it into a printable file make the whole process labor and time intensive. Many orthopedic applications of 3DP do not require immediate availability of the model. In orthopedic trauma, however, fractures have to be treated in a matter of days. These strict time constraints limit the use of an outside supplier and favor in-house production if the processing of the data is not too time-consuming and can be carried out by the staff. While many printers and materials are readily available at very low cost, software solutions are either very expensive or require excess manual labor that is too cumbersome for daily use.

### Related works and aim of the study

Acetabular fracture treatment often involves a complex 3D fracture pattern. Being a complex procedure, it requires skill and good preparation, making it an ideal candidate for being supported by 3D printing. Brown et al. published the first studies of 3D printing in acetabular fracture surgery [5, 6]. In the past years, additional authors have published clinical results, showing first evidence that it is an effective method to treat those patients [7, 8]. However, little detailed information has been shared on how to derive a useful fracture model from an initial CT DICOM dataset and the effort it takes.

Three main benefits are expected from using a 3D printed fracture model: Firstly, the fracture can be haptically explored in an ergonomic manner, improving understanding of the pathology [9]. Secondly, the fracture reduction strategy can be planned [10]. Thirdly, osteosynthesis plates can be bent to the anatomy and bridging the fracture. After re-sterilization, they can intraoperatively be used as a reduction guide. As a result, this saves intraoperative bending time, potentially improves implant fit [7] and perhaps even facilitates reduction to the shape of the plate.

The first benefit seems easily achievable, and studies have compared the potential of the 3D model when comparing it to 3D CT renderings on a screen [11]. To achieve the second benefit, the model should ideally allow the reduction in fractures. This requires the 3D model to be slightly flexible, have clearly separated fragments and cleaned artifact-free fracture lines allowing the model fragments to be bent into their place. Creating such a model is significantly more labor intensive than an un-separated, rigid model. These two steps are a requirement for the final goal which is pre-bending the plates. This is why many authors chose to use the “mirror technique,” mirroring the non-injured side to obtain a fractureless model. However, there are two downsides of this method: Firstly, the surface of the mirrored side may not perfectly fit to its counterpart; secondly, because it does not display the fracture lines, it cannot adequately be used to develop a surgical strategy and may result in impaired reduction and implant placement [12]. The “ideal model,” providing all those three aforementioned features in combination, has not yet been described in the literature.

Furthermore, the existing literature is rather limited in regard to detailed methodology and time requirements for the process of model creation [12]. Many authors use commercial software such as Mimics Innovation Suite (Materialise) [6, 7, 13], associated with very high cost. Others use open-source software such as 3D Slicer [14] and even more use a combination of software to create the printer file [1]. The printing itself is carried out either on PolyJet, selective laser sintering (SLS) or fused deposition modeling (FDM) printers, with only the latter being accessible without a significant capital investment.

In short, there is no established process for 3D in-house printing of acetabular fracture models that is not prohibitively labor intensive to be used in daily practice. The current available literature shows that a method meeting the aforementioned requirements does not yet exist. Thus, it is the aim of this work to provide a solution and validate it on a cohort of patients. Focus is set on process parameters such as time and cost, as well as on clinical parameters in terms of operation time, blood loss and final outcome.

**Fig. 1:**
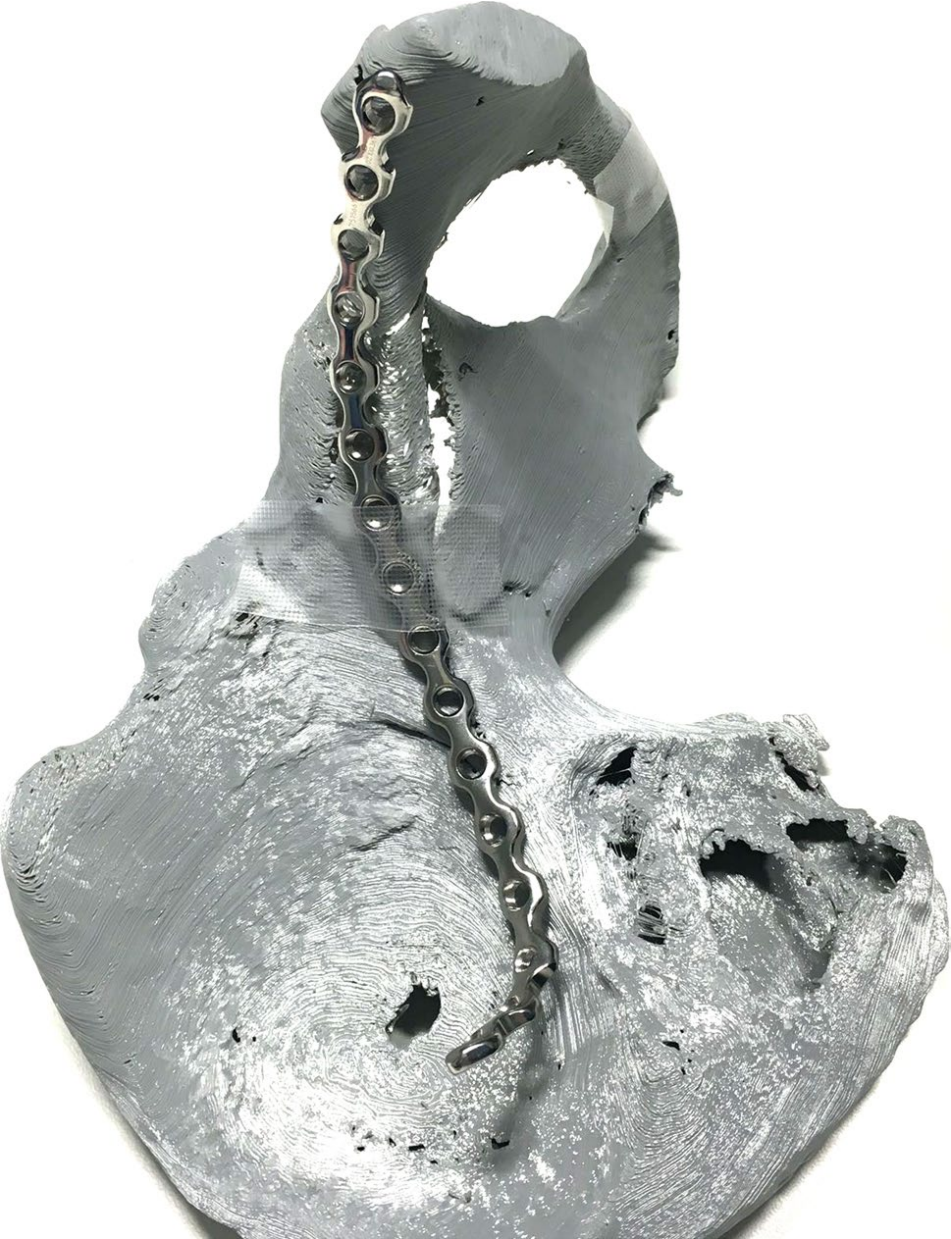
Steel reconstruction plate pre-bent to the acetabular fracture model prior to surgery. The implant was sterilized overnight for next-day surgery

In order to create a solution for the routine treatment of acetabular fractures, the following requirements were agreed among the authors (Fig. 1):

- The final 3D printed model has to be available within 24 h after making the treatment decision
- The model has to be shaped according to the anatomy seen on CT, and the relevant fracture parts have to be clearly visible on the model
- Reduction in the fractures as well as pre-contouring the plates has to be possible

Our hypotheses were the following:

- It is possible to provide a semiautomatic method to create printable files from acetabular fracture CT data and show clinical feasibility on a cohort of patients.
- One can produce models of a quality that render intraoperative plate bending obsolete, and it is possible to quantify the clinical and technical results.

## Materials and methods

### Patient collective and image data acquisition

This retrospective analysis was performed on data generated while observing consecutive patients of our level 1 trauma center, who were surgically treated for acetabular fractures. After surgical treatment was decided, CT image data were extracted from a Siemens SOMATOM Force dual-source MDCT using a soft tissue kernel (BR32D) ideally with a submillimeter slice thickness (0.5–1.25 mm).

The DICOM data were then transferred to an Apple MacBook Pro 2017 (3.1 GHz Intel Core i7, Radeon Pro 560 4 GB) for further processing. The 3D mesh model was generated by a custom-made extension [15] for 3D Slicer (https://www.slicer.org) [16], an open-source software, which integrates image and polydata libraries. The software extension was programmed to generate a printable model by carrying out the following tasks:

### Segmentation

**Fig. 2:**
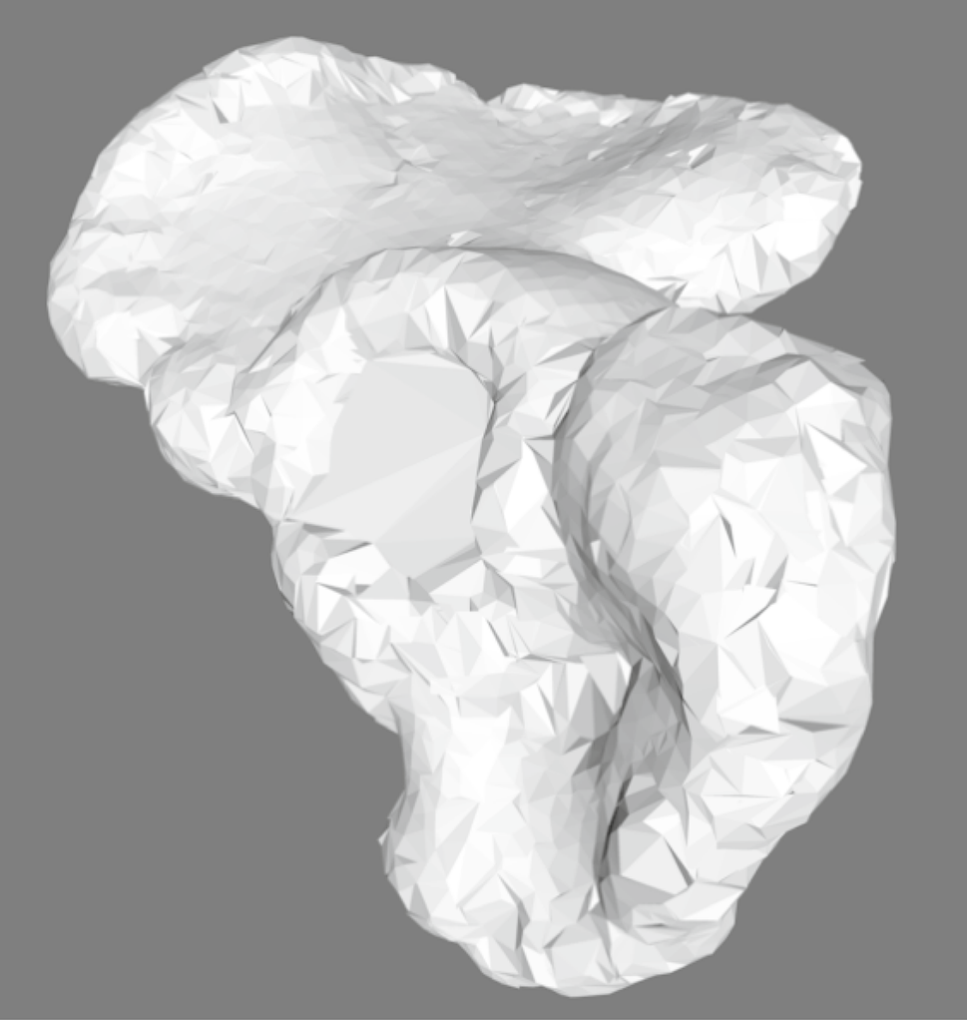
Result after the first shrinkwrap iteration: large faces, covering the acetabular cup, are visible

Segmentation was carried out using Slicer-integrated tools. A common threshold operation was applied using a lower cutoff between 200 and 350 Hounsfield Units depending on the bone density and fracture configuration. In most cases, minimal manual editing was needed for separating pelvis, femoral head and the sacrum. Subsequently, the pelvis was separated from the femoral bone, the opposing hemipelvis bone, the sacral bone and other artefacts resulting in a roughly segmented singular hemipelvis bone (“segmented model”) (Fig. 2, green line/areas). The time needed for the manual process was recorded.

### Surface filtering

**Fig. 3:**
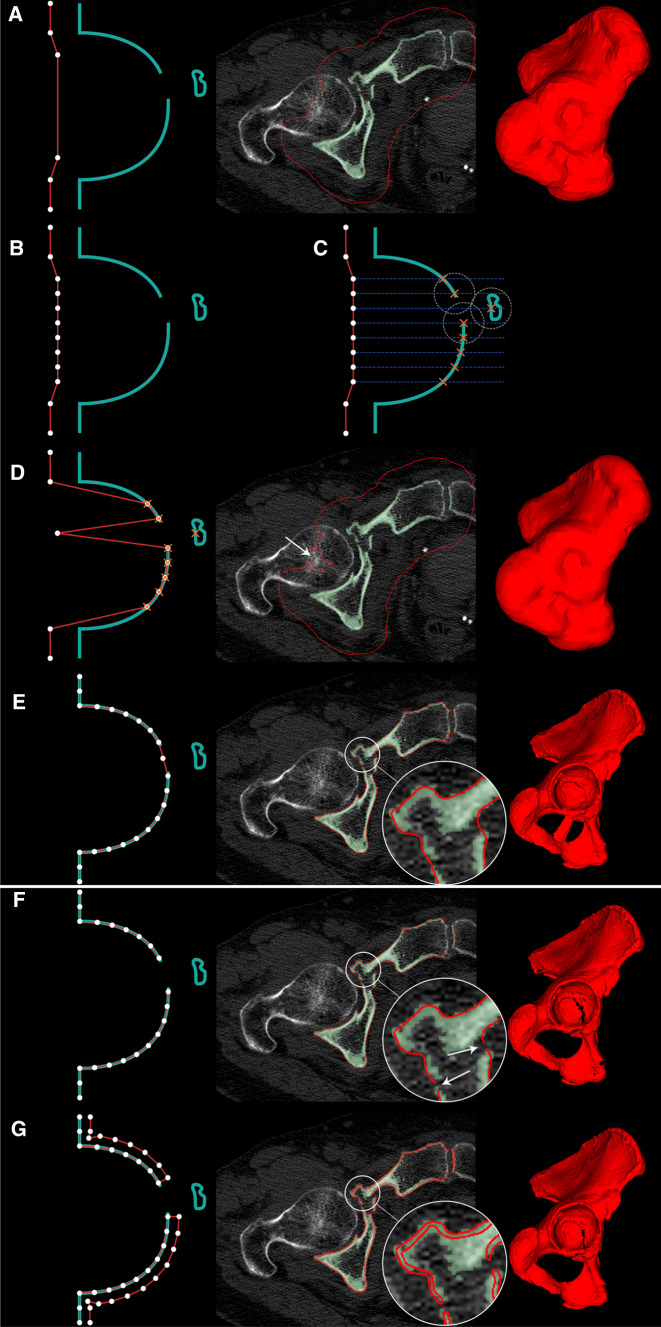
Filtering result separated in its interim stages. Red: surface model. Green: segmented model. White dots: vertices of surface model. Blue Line: rays with configurable length hitting segmented model. Orange cross: intersection points. Gray circle: example of the intersection point distance threshold to other intersection points. A: after the first shrinkwrap loop, the sphere adapted to the segmented model keeping a defined distance. B: after the raycast, some vertices are projected in deeper holes like the acetabular cup. C: after subdivision of long edged faces. D: raycast. E: result of the second shrinkwrap loop. F: after deletion of redundant vertices not close to the segmented model, resulting in a non-solid model. G: solidified surface model, ready to print

Since the printed models are used for the pre-contouring of reduction plates, mainly the cortical bone surface is required to be printed. After a threshold, cartilage bone as well as cortical parts with irregular thickness and artifacts is segmented, resulting in unnecessary prolonged printing time. Hence, to eliminate these non-needed areas, the following filtering pipeline was applied. It results in a hollow surface mesh (“surface model”) as shown in Figs. 2 and 3 as a red line. Experimentally determined default settings of the filter and symbols used in the mathematical description are listed in Table 1.

**Table 1:** Default settings used for the surface filtering pipeline in this paper

Mathematical symbol	Description	Default setting	Representation in Fig. 3
*O*	Initial shrinkwrap offset	15 mm	A, distance between red and blue lines
*E*_*T*_	Threshold edge length to determine if the corresponding face is used for the projection step or not	20 mm	A
*E*_*S*_	Resulting maximal edge length of subdivision faces	5 mm	C, distance between vertices and conclusively between blue lines
*P*_max_, *P*_min_	Maximal and minimal projection length between surface and segmented model	0 mm, 100 mm	C, length of blue line
*D*	Maximal allowed distance between the nearest neighbor projected vertices	2 mm	C, radius of gray circle
*G*	Maximal allowed gap between surface and shrinkwrap model after the final shrinkwrap	1 mm	F
*T*	Model thickness of the solidification process	0.8 mm	G

1. Bottino et al. [17] described the shrinkwrap as an algorithm to extract a polygonal mesh from an isosurface. An iterative combination of a shrinking process followed by a remeshing process wraps a sphere to an isosurface. This approach is used and extended by Kobbelt et al. [18] for surface refinement of an already existing polygonal mesh. Our method is used for surface refinement as well; however, fracture lines and deep holes like the acetabular cup are preserved, which is not the case for Kobbelt et al. and other shrinkwrap methods.
  Our algorithm starts with a spherical mesh $S$ (“surface model”), which is initialized around the target mesh $M$ (“segmented model”). For every vertex position $s \in {\mathbb{R}}^{3}$ on $S$, a corresponding closest point $m \in {\mathbb{R}}^{3}$ on $M$ is searched. $\overrightarrow {{{\text{dir}}_{nn} }}$ constitutes the normalized direction vector, which connects both closest points, and $\lambda$ is their distance. The shrinking operation of $S$ works as follows:
  $$ s_{t + 1} = s_{t} + (\lambda - O)\overrightarrow {{{\text{dir}}_{nn} }} , $$
  where $O$ is the offset kept between both meshes (Table 1).
  After that, the model gets remeshed via a voxelization of the surface model $M$ and subsequently applying a marching cubes algorithm for the conversion back into a surface mesh with equally distributed vertices. This step is illustrated in Fig. 3A. Both operations, shrinking and remeshing, are iterated 3 times.
2. However, since the shrinkwrap uses a minimal vertex distance approach, it fails, especially for regions like the acetabular cup. Anatomically, it is deeper than its rim is wide; therefore, the algorithm will always find the closest surface on the rim, not the bottom of the cup (Fig. 2). To address this issue, a one-time projection step was introduced after the shrinkwrap step as described in the following (Fig. 3B–D):
  - All faces with an edge longer than E are separated from the surface model, resulting in a new model $S_{\text{sub}}$. A Loop Subdivision Algorithm, as described by Pakdel [19], is applied to $S_{\text{sub}}$, subdividing them into smaller triangles to a maximal edge length of *E*_*S*_ (Table 1, Fig. 3B).
  - For each new vertex position $s \in {\mathbb{R}}^{3}$ on $S_{\text{sub}}$, its normal vector $n \in {\mathbb{R}}^{3}$ is calculated. After that, a projection intersection point on $M$ is searched for every $s$ on $S_{\text{sub}}$. $\lambda$ constitutes the distance between origin and intersection point:
    $$ s^{p} = s + \lambda n $$
  - The vertex is moved to the new position $s^{p}$ (Fig. 3D), if both the following conditions are true:
    $P_{\min}  < \lambda < P_{\max}$ to reduce failed projections to far off mesh elements as well as speed up.
    *d *< D with
      $$ d_{i} = \mathop {\hbox{min} }\limits_{k,k \ne i} \left( {\left\| {s_{i}^{p} - s_{k}^{p} } \right\|} \right), $$
      where $i$ is the index of the surface vertex. This is performed to prune isolated projections that most often hit elements inside small gaps, like fissures inside the acetabular cup (Fig. 3C).
  - Finally, $S_{\text{sub}}$ is reattached to $S$.
3. Other 7 shrinkwrap iterations are performed; the algorithm is applied without any offset to smooth out the projection artefacts and fully adhere to the surface model to the segmented model and eliminating any remaining distance between the surfaces (Fig. 3E).
4. For every vertex position $s$ on $S$, their closest distance to $M$ is calculated, with $m$ being the closest point:
  $$ g = \hbox{min} \left( {\left\| {s - m} \right\|} \right) $$
  If $g \ge G$, the corresponding vertex gets deleted. This results in a non-manifold model with visible fracture lines as shown in Fig. 3F.
5. Finally, the currently non-manifold model gets solidified, applying a thickness $T$. For every vertex position $s$ on $S$, the corresponding normal vector $n \in {\mathbb{R}}^{3}$ is calculated. Then, a new corresponding vertex $s_{\text{copy}}$ is created at:
  $$ s_{\text{copy}} = s + nT, $$
  where *T* is the desired thickness of the resulting mesh (Table 1).

Connecting all new vertices with faces and also closing edges between the newly generated isosurface and $S$ finally result in a manifold model as shown in Fig. 3G.

**Fig. 4:**
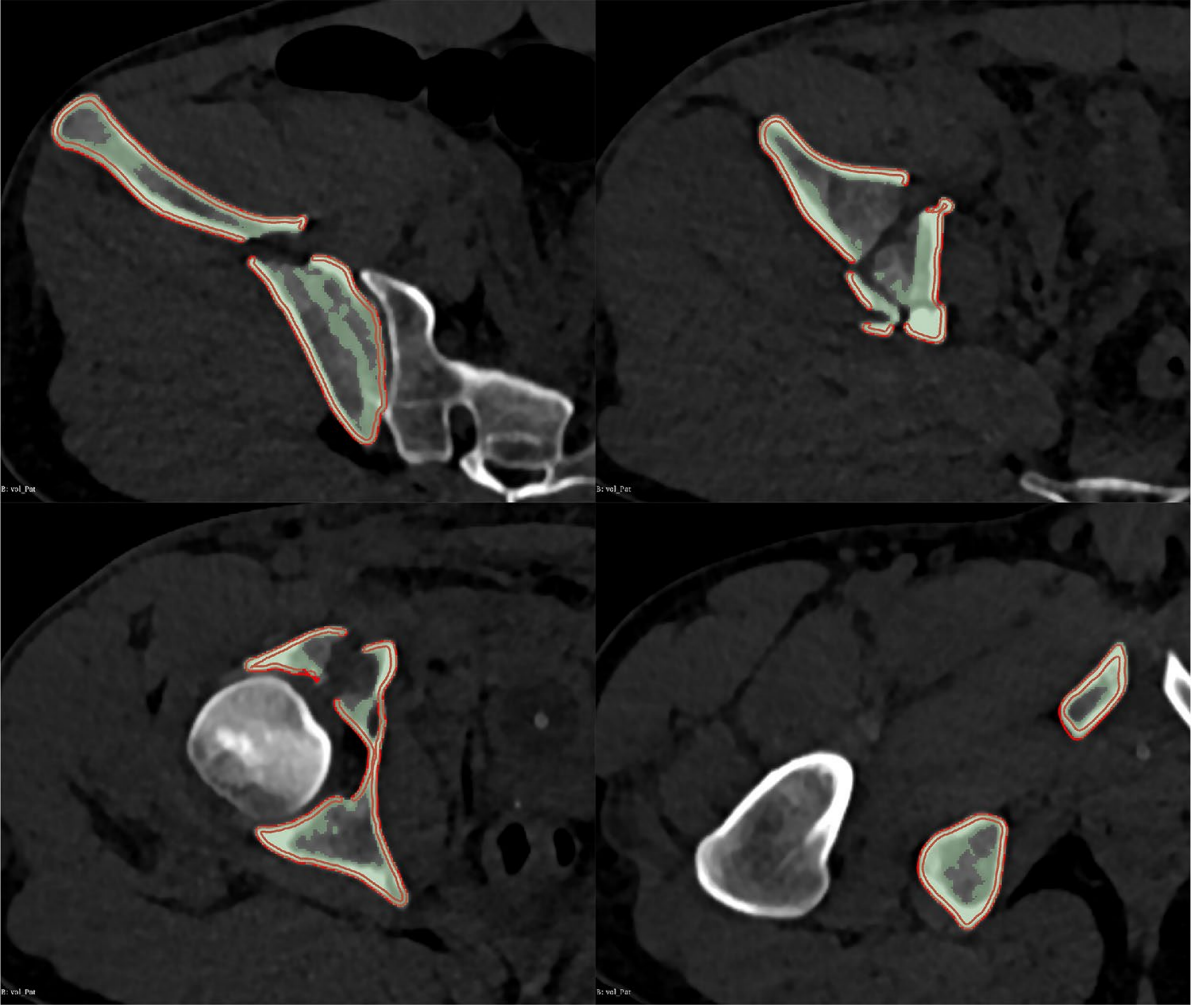
Result of the filtering process, axial ct slides. Used for verification of the filter result. Green: original threshold segmentation. Red: resulting surface model

The time needed for software processing was recorded for each case. Also, the resulting model was inspected carefully and compared to the patient’s CT. An overlapping view as shown in Fig. 4 was used for this verification process. The principally used default settings were adjusted for a better filter result if needed.

### 3D printing

All surface models were further processed and sliced using the Ultimaker Cura software and printed with an Ultimaker 2 + with a modified feeder using PLA material.

The models were placed on the print bed with the iliac fossa facing the bed and the iliac crest and the tuberosity of the ischium touching the bed. Moreover, the longest model axis was aligned with the support lines direction. All cases were printed with the following print settings: travel speed 150 mm/s, print speed 60 mm/s, infill density 100%, layer height 0.2 mm, and support structures were printed as lines with a density of 5% for structures with an overhang of 70°. The alignment and slicing time were measured as well as the printing time itself.

The printing time of the original models, without the surface filter applied, was calculated. Accordingly, the models were placed in the same positions and rotations as the filtered ones; also the same printing settings were used. Hence, the printing times could be compared.

### Validation of the 3D printed models

After 3D printing, all models were CT scanned by a Siemens SOMATOM Force dual-source MDCT. The images were extracted using a soft tissue kernel (BR32D) and a slice thickness of 0.75 mm. After segmentation using a threshold with range between about − 900 and − 200 HU matching the PLA density, the filtering pipeline was used on this segmentation with the same settings as for the corresponding patient.

The resulting surface models were registered using a landmark registration method by VTK (Visualization Toolkit). For every model, 10 landmarks were used. RMS-Error for the registration, as well as the surface distance of each vertex of the printed surface filtered model to the computed model, was calculated.

### Plate pre-bending and sterilization process

To prepare for surgery, the fractures on the model itself were reduced. In most cases, cutting connective structures was obligatory to enable full fracture reduction. Since wall thickness was 0.8 mm, this could be carried out by a heated wire cutter (Conmed SmartPin cutter). The model reduction time was recorded.

Subsequently, the reconstruction plates were pre-bent for later implantation as it would be done during surgery without the model. In all cases, 14-hole steel reconstruction plates (DePuy Synthes) were used. Depending on the urgency and capacity of early treatment, the plates were either bent unsterile a day before surgery and re-sterilised or were kept sterile during surgery by putting the models in a sterile plastic bag. The plate bending time was also recorded.

### Surgical procedure

For the anterior and posterior acetabular column, plate and screw fixations were performed. Access to the anterior column was made via the Stoppa approach. The posterior column was treated by the Kocher–Langenbeck approach. In the Stoppa approach, an incision in the midline of the abdomen provides access to the anterior pelvic ring and acetabular column. The posterior Kocher–Langenbeck approach is more extensive since hip rotators (piriformis muscle, etc.) must be detached to reach the posterior acetabular column.

Intraoperative blood loss, operation time, as well as intraoperative reduction and plate bending time, were recorded.

### Clinical follow-up

Patients were admitted to our outpatient department for follow-up. At an average of 12 months, outcome was assessed by Harris Hip Score, modified Harris Hip Score and Merle d’Aubigne Score. Informed consent was obtained from all participants included in the study. In addition, approval for this study was obtained by the local ethics committee.

### Data analysis

SPSS 17.0 (IBM) was used to create boxplots and to calculate standard deviation and mean values of the data. VTK and NumPy were used for calculating model distances and registration.

## Results

A total of 12 cases were included in this study. The average age of the patients was 41.2 years, with 31% of the patients being women and 69% being men.

In 81% of cases, the operation was performed via the Stoppa approach, with only 18% of cases being the Kocher–Langenbeck approach. The mean operation time was 3:16 h, and the blood loss was 853 ml on average.

The mean of the “model creation time” was 11 min and 8 s. Three of the 12 patient hemipelvices were printed as incomplete, cropped models which reduced printing time accordingly.

The default parameters of the filtering pipeline were used for 11 of the 12 patients. The remaining case had to be adjusted slightly to achieve a better surface filtering result.

**Fig. 5:**
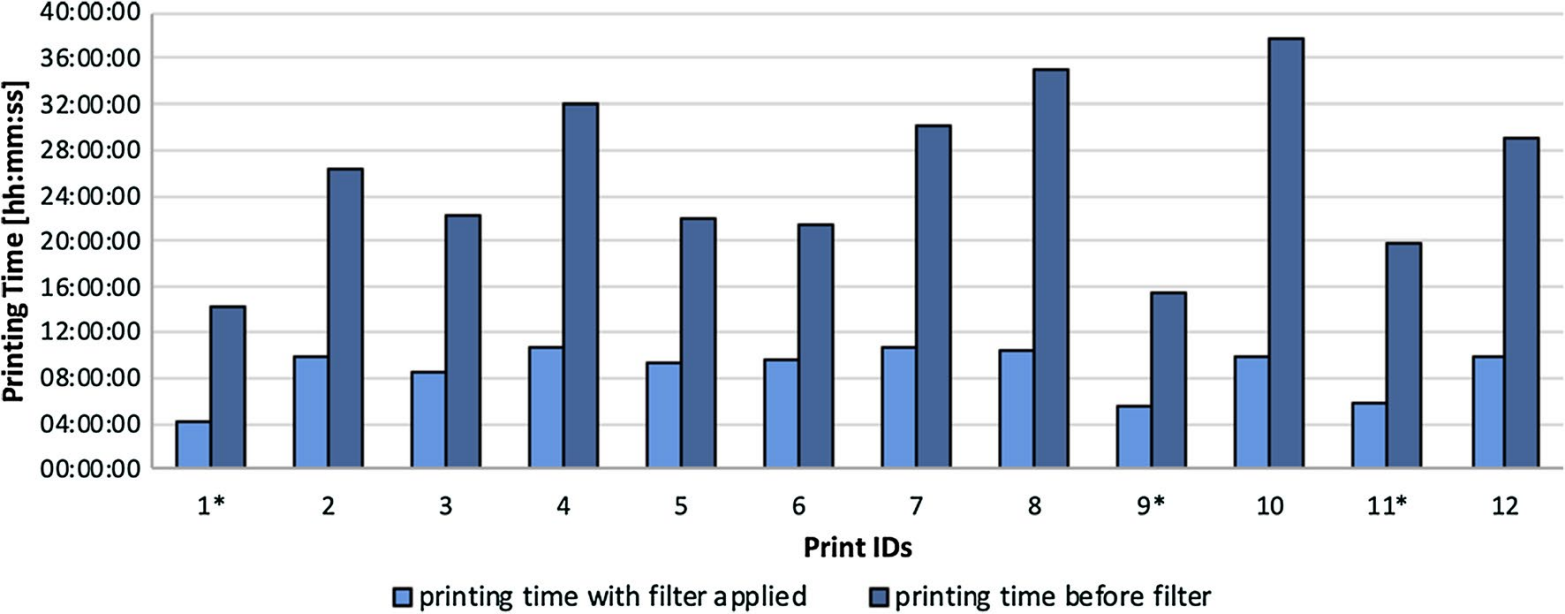
Calculated printing times before and after applying the filter for all 12 cases. *Cropped hemipelvises

The mean printing time was 8:40 h, excluding the cropped models at 9:50 h (Fig. 5). In comparison, the calculated mean printing time without applying the surface filter was 25:26 h, excluding that the cropped models had 28:24 h. This concludes an average printing time reduction of 65%.

**Fig. 6:**
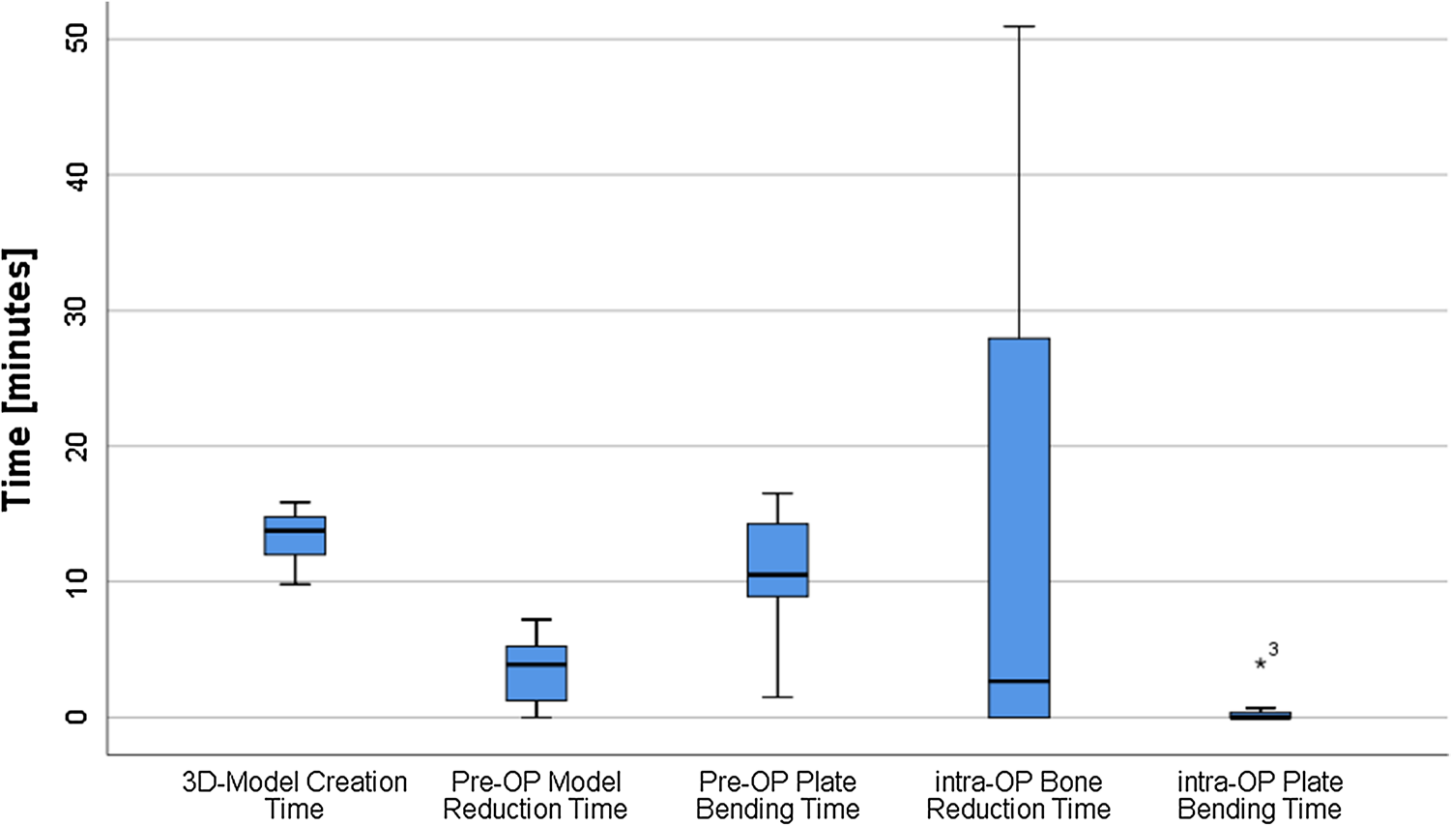
Time required for model creation, reduction and plate bending

The average “pre-OP model reduction time” was 5 min and 30 s, and “pre-OP plate bending time” took 10 min and 18 s. The mean reduction time of the fracture fragments required during the operation was 12 min and 42 s, whereas only 26 s was needed on average to further intraoperatively adjust the pre-bent plate, displayed as “intra-OP plate bending time” (Fig. 6).

The mean average deviation (MAD) of 10 of the 11 printed models compared to the computed model was under 1 mm with the largest maximal deviation of 19 mm for patient 9, which turned out to be a loose leftover support structure. However, overall no clinically relevant deviations were found (Table 2).

**Table 2:** Surface distances between the preprint model and printed model

Patient ID	RMS-error (mm)	*N* (vertices)	MAD [mm]	STD [mm]	MAX [mm]
1*	1.97	35,126	0.73	0.48	3.97
2	3.07	78,486	0.75	0.60	5.95
3	0.10	66,896	0.68	0.44	3.23
4	2.53	80,079	1.06	0.82	4.93
5	2.14	68,641	0.93	0.74	3.77
6	1.01	71,077	0.74	0.43	6.53
7	2.30	73,854	0.87	0.75	10.97
8	1.17	81,882	0.67	0.35	1.96
9	3.15	44,834	0.92	0.87	19.56
10	1.64	78,261	0.88	0.7	6.79
11*	1.15	42,124	0.67	0.53	5.15
12	2.91	77,231	0.71	0.54	5.02

For every vertex of the preprint model surface, the closest distance to the printed model surface was calculated

*Cropped hemipelvises

*RMS-error* root mean square error of the landmark registration, *MAD* mean average distance of all vertices, *STD* standard deviation, *MAX* maximal deviation of all vertices

**Fig. 7:**
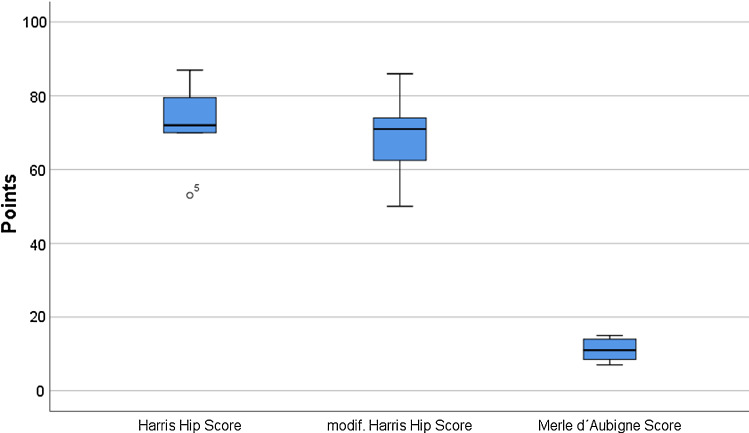
Mean hip function outcome scores at 1 year (points)

After 12 months, 9 patients underwent a structured follow-up. On average, the Harris Hip Score was 75.7 points, the Modified Harris Hip Score 71.6 points and the Merle d’Aubigne Score 11.1 points (Fig. 7).

The gradings were:

- (Modified) Harris Hip Scores < 70: poor results, 70–79: fair results, 80–89: good results, 90–100: excellent results.
- Merle d’Aubigne Score: < 12: poor results, 12–14: fair results, 15–17: good results, 18: excellent results.

## Discussion

In this study, we defined specific goals that had to be met to successfully evaluate a technology that supports acetabular fracture treatment with the help of 3D printing. We demonstrated the feasibility of a workflow incorporating in-house 3D printed acetabular fracture models for use in surgery. A novel surface filtering pipeline allowed to considerably reduce printing time and cost compared to recent publications, while allowing fracture reduction on the model itself. It is possible to have the implant bent to the model within 24 h after obtaining the CT dataset and either use a re-sterilized plate or bent it intraoperatively on the model protected by a sterile bag. With drastically reduced model creation and printing times, next-day acetabular fracture surgery with the help of 3D printing becomes possible.

The printing time could be reduced by about 65%. As shown in Fig. 3, superfluous cancellous structures inside the fractured bones are deleted. Moreover, very narrow cortical bone segmentations are solidified, leading to an equal shell thickness of the computed model. This major improvement is achieved by optimizing the following factors:

- Printing a model with an even wall thickness is ideal for the FDM printing technique as the material can be distributed evenly layer per layer in a circular manner. In contrast, an irregular wall causes the printer to process pieces of the wall separately, leading to a prolonged printing time.
- Furthermore, segmented artefacts of cartilage bone are causing the printer to build an extensively larger amount of support structures, hence prolonging printing time.

However, this solidifying process may lead to errors by very narrow bone pieces. In some cases, the iliac fossa showed both cortical sides being closer together than the solidifying thickness, leading to imprecision in these areas.

**Fig. 8:**
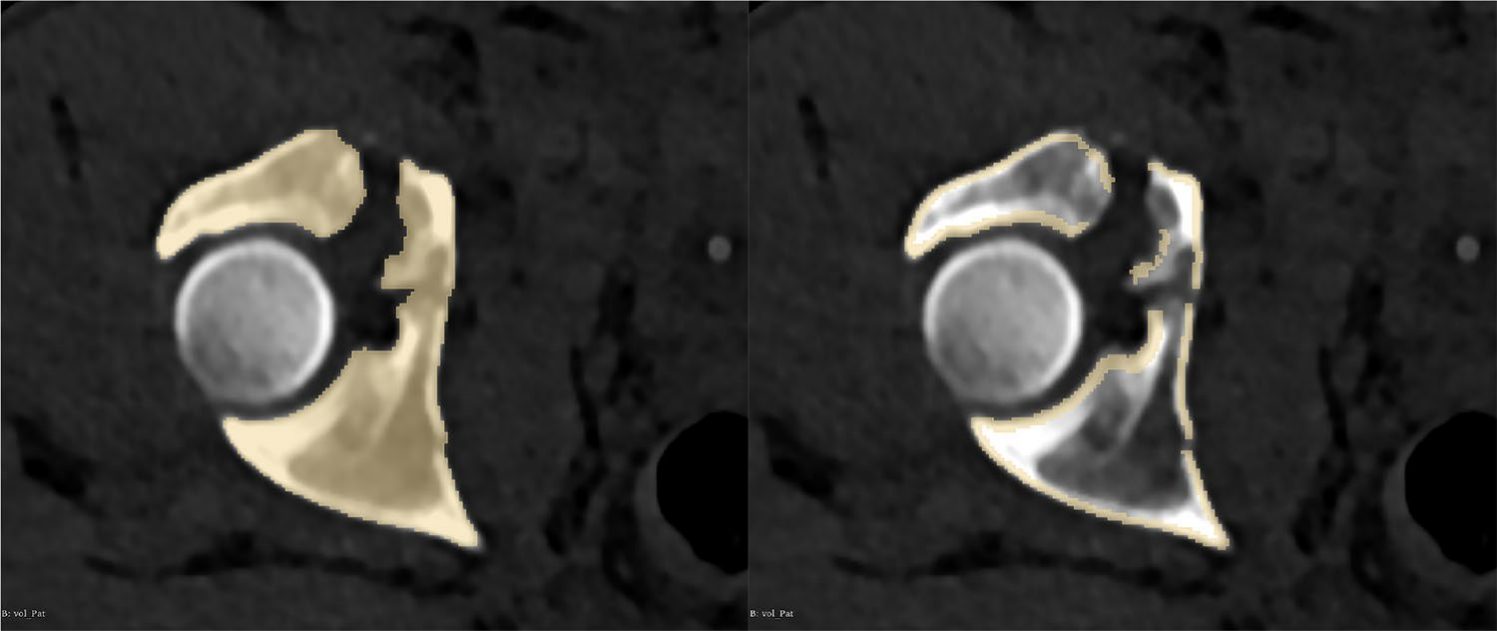
Comparison of the filtering pipeline with Growing from Seed techniques. Left: result of Growing from Seed Segmentation. Right: basic threshold and result using the filtering pipeline

As a foundation, a basic threshold is used for this workflow, since this method is very precisely conserving fracture lines in the cortical bone. For non-fractured bones, algorithms such as Grow From Seed or Watershed are mostly used for complete segmentation of the bone and afterward printing the models with low infill density for speeding up the print. This, however, is not feasible for fractured bones surfaces as those algorithms tend to cover fracture gaps as shown in Fig. 8. In contrast, our approach conserves fracture lines and makes it even possible to reduce fractures on the model.

Besides proving the feasibility of our method using custom-made software, the use of pre-fit reconstruction plates as well as printed models seemed absolutely valuable for experienced and inexperienced surgeons in terms of fracture understanding. The outcome of our small patient cohort corresponds to the results of Maini et al. [7], showing good alignment, short surgery time and little blood loss. Our mixed follow-up results (see the clinical scores) may most likely be caused by the severity of the patients injuries in the first place; however, more studies with larger cohorts are needed for the final evaluation.

A submillimeter CT scan of the patient’s facture, reconstructed using a soft tissue kernel, is a requirement of this method. Those datasets can usually be generated by modern CT scanners using standard scanning parameters. Still, the segmentation results are not ideal in every case, as narrow fracture lines and some displaced fractures may remain obscured. Therefore, the models should not be used for diagnostic purposes and should always be evaluated in accordance with the initial CT dataset. However, first validation results are showing a sufficient accuracy of the models for being used for aligning the plates [20].

Contrary to most other software solutions such as the “mirror technique,” this method creates models without most of the cancellous structures, thus enabling fracture reduction. With an efficient method of model creation as shown, plate bending on the intraoperative situs becomes obsolete for most cases, saving time and effort by transferring this task to a safer and more convenient preoperative environment. However, in 2 cases some extent of intraoperative correction of the shape of the plate was required. Like others, for these cases pre-bending was not performed by the leading surgeon of the procedure, which resulted in slight changes of intended plate placement. Moreover, a learning curve effect can be expected that will reduce corrective bending even more over time.

The material cost associated with each case was below 5€, and the capital investment for a printer priced at less than 2500€. With the data processing potentially being carried out by existing staff members, the financial break-even for the investment can be obtained within a few cases, not taking into account the potential improvement in treatment. Moreover, the printer allows for further use cases. The current software has potential to be transferable to other surgical treatment areas; however, this needs to be evaluated in the future.

The demonstrated method can be applied in practically every hospital setting for acetabular fracture surgery, given the minimal investments for a 3D printer and filament. In our setting, surgeons and students could perform the model creation as well the printing after a training period by themselves with minimal time investment as shown in Fig. 6. However, printer maintenance possibly leads to further expenses in the future. Furthermore, in other settings extra technical employees might be necessary.

The filter pipeline is available as a 3D Slicer module for off-label use, downloadable in the integrated Extension Manager [15].

## Conclusions

We presented the first clinically practical technique for routine use of 3D printing in acetabular fracture surgery. By introducing a new filtering pipeline, we reduced printing time and cost compared to current literature and the state of the art. The goals set by the authors in the introduction were all met with the exception of the rare occurrence of the plate bending intraoperatively. By comparing the filtering results with the initial CT as well as with the CT of the resulting 3DP model, the quality of the result could be assured. In order to show a clear influence on clinical outcome, a larger prospective and controlled study would be desirable. For this, we share our application with the public for off-label use. It can be downloaded from Github [15].
