# Correction to: 3D printing method for next‑day acetabular fracture surgery using a surface filtering pipeline: feasibility and 1‑year clinical results

**DOI:** 10.1007/s11548-021-02348-7

**Published:** 2021-03-23

**Authors:** Simon Weidert, Sebastian Andress, Christoph Linhart, Eduardo M. Suero, Axel Greiner, Wolfgang Böcker, Christian Kammerlander, Christopher A. Becker

**Affiliations:** Department of General, Trauma and Reconstructive Surgery, University Hospital, LMU Munich, Campus Großhadern, Marchioninistr. 15, 81377 Munich, Germany

## Correction to: International Journal of Computer Assisted Radiology and Surgery (2020) 15:565–575 10.1007/s11548-019-02110-0

The article 3D printing method for next-day acetabular fracture surgery using a surface filtering pipeline: feasibility and 1-year clinical results, written by Simon Weidert, Sebastian Andress, Christoph Linhart, Eduardo M. Suero, Axel Greiner, Wolfgang Böcker, Christian Kammerlander and Christopher A. Becker, was originally published Online First without Open Access. After publication in volume 15, issue 3, page 565–575 the author decided to opt for Open Choice and to make the article an Open Access publication. Therefore, the copyright of the article has been changed to © The Author(s) 2021 and this article is licensed under a Creative Commons Attribution 4.0 International License, which permits use, sharing, adaptation, distribution and reproduction in any medium or format, as long as you give appropriate credit to the original author(s) and the source, provide a link to the Creative Commons licence, and indicate if changes were made. The images or other third party material in this article are included in the article’s Creative Commons licence, unless indicated otherwise in a credit line to the material. If material is not included in the article’s Creative Commons licence and your intended use is not permitted by statutory regulation or exceeds the permitted use, you will need to obtain permission directly from the copyright holder. To view a copy of this licence, visit http://creativecommons.org/licenses/by/4.0/.

The original article has been corrected.

